# Semi‐Automated Multi‐Label Classification of Autistic Mannerisms by Machine Learning on Post Hoc Skeletal Tracking

**DOI:** 10.1002/aur.70020

**Published:** 2025-03-14

**Authors:** Christian Lemler, Solvejg K. Kleber, Leonie Polzer, Naisan Raji, Janina Kitzerow‐Cleven, Ziyon Kim, Simeon Platte, Christine M. Freitag, Nico Bast

**Affiliations:** ^1^ Department of Child and Adolescent Psychiatry, Psychosomatics and Psychotherapy, Autism Research and Intervention Center of Excellence University Hospital Frankfurt, Goethe‐University Frankfurt am Main Germany

**Keywords:** autism spectrum disorder, machine learning, mannerisms, multi‐label classification, stereotypic behavior

## Abstract

Mannerisms describe repetitive or unconventional body movements like arm flapping. These movements are early markers of restricted and repetitive behaviors (RRBs) in autism spectrum disorder (ASD). However, assessing mannerisms reliably is challenging. Even after extensive training in behavioral observations, inter‐rater agreements for mannerism items remain insufficient. The current study used machine learning (ML) to classify mannerisms from videotaped behavioral observations in children with ASD. We developed a classification scheme for mannerisms as ground truth and applied it to videotaped behavioral observations from an early intervention study. ML was used in two steps: First, the OpenPose algorithm post hoc extracted features based on body movements in the videos. Second, a long short‐term memory (LSTM) neural network classified the features in a multi‐label approach to distinguish between the absence of mannerisms, flapping, jumping, and both flapping + jumping. The trained models achieved 70.2% accuracy (*F*1 score: 31.8%) using nested cross‐validation. The analysis improves on previous videotaped ML classification studies by splitting training and test data subject‐wise, highlighting its clinical applicability. The LSTM models are made publicly available for use with other video datasets. Our results show that ML‐based classification of mannerisms is a promising tool for enhancing objective diagnostic methods of behavioral observations.

**Summary**

- The current study applied artificial intelligence (AI) to videos of children with autism spectrum disorder (ASD).
- The algorithm learned to distinguish between autistic movements like flapping and jumping.
- This could improve diagnostic procedures in future clinical practices.

Autism spectrum disorder (ASD) is a heterogeneous neurodevelopmental condition. It is characterized by difficulties in social interaction and communication, as well as restricted and repetitive behavior (RRB) (DSM‐5; American Psychiatric Association 2013). The diagnostic gold standard combines a parental interview with a behavioral observation (Falkmer et al. 2013). This process is time‐consuming and personnel‐intensive (Kamp‐Becker et al. 2018). Limited personnel capacities may contribute to a late initial ASD diagnosis at the mean age of 5 years (Brett et al. 2016). However, earlier intervention is linked to greater symptom reduction (Robain et al. 2020) and might reduce the individual's lifetime burden (Peters‐Scheffer et al. 2012). Therefore, early diagnosis is crucial. RRBs are early symptoms (Elison et al. 2014) and a core diagnostic feature of ASD (Chaxiong et al. 2022). Identifying RRBs objectively in young children could support an earlier diagnosis of ASD.

Video‐recorded behavioral observations provide objective information on RRBs (Zwaigenbaum et al. 2005). Currently, these observations are manually coded using different instruments, which are clinically demanding (Luallin et al. 2022). Additionally, inter‐rater agreement for RRBs tends to be low to moderate (Janvier et al. 2022). For specific behaviors like *mannerisms*, manual coding showed particularly low reliabilities (Carruthers et al. 2021). We propose that machine learning (ML) offers a more efficient and cost‐effective approach to identifying RRB symptoms, especially mannerisms, in videotaped behavioral observations.

ML is a subfield of Artificial intelligence (AI) that utilizes algorithms to mimic data‐based learning. ML advanced significantly through deep learning (Goodfellow et al. 2016), which uses artificial neural networks (ANNs) that simulate neuronal information processing to uncover patterns in data. A convolutional neural network (CNN) is a type of ANN designed to process grid‐like data structures, such as images or videos. CNNs are particularly effective in image recognition tasks (Pak and Kim 2017), but their inputs are independent. In contrast, long short‐term memory (LSTM) networks have memory cells that preserve information across the network, making them well suited for time‐series data (Hochreiter and Schmidhuber 1997). In classification tasks, LSTMs outperform traditional methods like decision tree algorithms (Bansal et al. 2022). Thus, LSTMs are a promising method for classifying mannerisms in video sequences of autistic children (Voulodimos et al. 2018).

In autism research, supervised ML has been applied to various data types, including genetic, imaging, and movement data (Hyde et al. 2019; Song et al. 2019). “Supervised” means that the algorithm is trained using labeled data, such as diagnostic labels like “ASD” and “non‐ASD.” The labeled data is split into training and test sets. During training, the algorithm builds a model to identify subjects with ASD. In testing, the retained data evaluates the model's performance using metrics like accuracy. Cross‐validation is commonly used to assess the model's generalization. In k‐fold cross‐validation, the data is divided into *k* subsets. For example, in 3‐fold cross‐validation, three subsets are tested, and an average performance is calculated. We combined supervised ML with k‐fold cross‐validation to develop a generalizable model for a semi‐automated classification of mannerisms from video data.

Previous research applied supervised ML to classify movements in autistic children (Table 1). Goodwin et al. (2011) used wrist and torso accelerometers to improve the classification in a study with six autistic children. This study was the only one to report inter‐rater reliability (IRR). Movements were labeled as “flapping” “rocking” or a combination of both “flap rock.” Non‐stereotypical movements (labeled as “unknown”) were randomly undersampled to balance the data. Undersampling frequent features is often used to prevent the algorithm from defining classes by occurrence likelihood. The accuracy for identifying mannerisms ranged from 82.5% to 96.4%. However, sensor‐based procedures require sensor attachments, which may not be well tolerated by participants. Additionally, these methods need a priori planning. In contrast, video recordings of behavioral observations are often available in healthcare institutions, allowing for a nonintrusive, post hoc identification of relevant features.

**TABLE 1:** Previous research using machine learning for the classification of autistic movements.

	Goodwin et al. (2011)	Vyas et al. (2019)	Cook et al. (2019)	(Zhang, Tian, et al. 2021)	Current study
ML architecture	Decision tree	CNN	Decision tree	LSTM	LSTM
Train‐test‐split	Subject‐wise^a^	Random (80/20)	Random (80/20)	Random (70/30)	Subject‐wise
Classification	Multi‐class	Binary	Binary	Multi‐class	Multi‐label
Feature extraction	Handcrafted	Mask R‐CNN	OpenPose/handcrafted (e.g., velocities)	OpenPose/handcrafted (e.g., velocities)	OpenPose
Open‐source	No	No	No	No	Yes
Data source	Accelerometers	Video	Video	Video	Video
Labels	Flapping, rocking, flap rock, unknown	Typical, atypical	Typical, atypical	Sit, stand, shake hands, shake the body, squat	Flapping, jumping, flapping and jumping, nothing
Validation	10‐fold CV	5‐fold CV	5‐fold CV	—	Nested 3‐fold CV
Sample size	6 ASD	—	35 atypical, 33 typical	—	52 ASD
Gender	6 male	—	—	—	39 male 13 female
Age range (years)	13–20	—	~3 to 12	~5 to 10	*M* = 4.13 SD = 0.86
ASD diagnostic	According to DSM‐IV‐TR	—	Not diagnosed	—	Gold standard^b^
Inter‐rater reliability (IRR)	*κ* = 0.32–0.95	Not reported	Not reported	Not reported	ICCs = 0.53–1.0^c^

Abbreviations: ASD, autism spectrum disorder; BOSCC, brief observation of social communication change; CNN, convolutional neural network; CV, cross‐validation; ICC, intraclass correlation coefficient; LSTM, Long short‐term memory; M, mean; RCT, randomized controlled trial; SD, standard deviation.

^a^ Limitations by using overlapping windows (Dehghani et al. 2019).

^b^ Autism diagnostic interview‐revised (ADI‐R), autism diagnostic observation schedule 2 (ADOS‐2) and clinical evaluation.

^c^ For items flapping and jumping.

Previous studies showed that CNN‐based methods are feasible for feature extraction from video data. Feature extraction can reduce raw input (e.g., video data) to a set of features (e.g., skeleton points) relevant to the classification algorithm. Vyas et al. (2019) used the pre‐trained pose estimator Mask R‐CNN to extract skeleton joints from 10‐s videos obtained from an autism home video database, which were labeled as “typical” and “atypical” behavior. After extensive preprocessing, the features were used in a supervised approach, with 80% of the data for training and 20% for testing. A CNN was applied for binary classification, achieving 72.4% accuracy (precision = 0.72, recall = 0.92) using 5‐fold cross‐validation. Cook et al. (2019) used YouTube videos from school‐aged children and applied the CNN‐based OpenPose algorithm for feature extraction. These features were used to classify “typical” and “atypical” behaviors from 68 videos (35 atypical, 33 typical). An 80/20 train‐test split and 5‐fold cross‐validation were used with a decision tree achieving an accuracy of 71% for binary classification. (Zhang, Tian, et al. 2021) applied OpenPose to extract skeleton key points and focused on movements such as “sit,” “stand,” “shake hands,” “shake the body,” and “squat.” They used 749 video sequences of autistic children, randomly splitting 70% for training and 30% for testing. Preprocessing included scaling key points, removing head key points, discarding frames with missing data, and using adjacent frames to calculate missing key points. An LSTM algorithm with softmax classification was then applied for multi‐class classification, achieving results between 90.3% accuracy for “shake the body” and 99.6% for “stand”. Overall, previous studies established the CNN‐based algorithm OpenPose as a promising method of feature extraction in video‐based classification.

Previous ML studies on movement identification using videos share some methodological limitations. They only identify singular movements in the training procedure. However, children often show more than one mannerism simultaneously. For example, a child may show “flapping” and “jumping” at the same time. This suggests the need for an algorithm trained on data that includes multiple movements occurring together, which more closely resembles real‐world behavior. Additionally, the definitions of stereotypic behavior were inconsistent across studies. Labels like “flapping,” “rocking,” “flap rock,” “sit,” “stand,” “shake hands,” “shake the body,” and “squat” had all been classified as stereotypic behavior, though actions like “sit” or “stand” are not related to the RRB domain. Furthermore, previous studies usually used a random split function, mixing sequences from individual participants into both training and test sets. This approach contradicts diagnostic needs, where the algorithm should be trained on a limited group of children and then applied to a new, different group of children. As a result, the generalizability of previous ML approaches is questionable, while splitting data by subject would enhance diagnostic applicability.

The current study applies ML to classify mannerisms from videotaped behavioral observations. It advances previous ML classification methods by (1) using multi‐label classification to identify different mannerisms simultaneously, (2) implementing a clinically driven mannerism classification scheme, and (3) applying a subject‐wise split, to enable the algorithm to identify mannerisms in new individuals. Additionally, the study uses an objective, open‐source method of feature extraction with minimal preprocessing. Thus, the resulting algorithm can be applied to existing repositories of videotaped behavioral observation in healthcare institutions around the globe.

## 1 Method

### 1.1 Participants

The sample was drawn from the baseline measurement of the multicenter randomized controlled trial for the Frankfurt Early Intervention Program (Kitzerow et al. 2020). The trial was approved by the local ethics committee (10/18). The final sample included 52 autistic preschoolers (Table 2). An ASD diagnosis was confirmed using gold standard diagnostics based on DSM‐5 criteria: the autism diagnostic interview‐revised (ADI‐R; Bölte et al. 2006), the autism diagnostic observation schedule 2 (ADOS‐2; Poustka et al. 2015), and clinical evaluation. Exclusion criteria were a nonverbal developmental quotient (DQ) or intelligence quotient (IQ) of 30 or lower, a nonverbal mental age of 12 months or less, diagnosed vision or hearing impairments that interfere with therapy, cerebral palsy, chronic neurological disorders, unstable epilepsy, neurodegenerative disorders, Rett/Angelman syndrome, a history of severe psychosocial deprivation, attachment disorders, institutional upbringing, and parents who were not verbally fluent in German or unable to read German.

**TABLE 2:** Sample description.

	*M*	SD
Age (months)	49.54	10.34
Test age (months)	26.02	16.79
ADI‐R total score	40.59	9.99
ADI‐R total (toddler)	19.61	3.63
ADOS‐2 calibrated severity score	7.40	1.76
ADOS‐2 social affect	7.10	1.84
ADOS‐2 RRB	7.75	1.77
ADOS‐2 item mannerisms	1.19	0.79
BOSCC total score	30.16	9.97
BOSCC social communication	20.93	7.43
BOSCC RRB	9.23	3.76
BOSCC item mannerisms	1.91	1.81
SRS‐16 total	27.46	7.71

*Note: N* = 52 (male = 39, female = 13). Test age was assessed with the Bayley Scales of Infant Development‐III (Bayley‐III) (Reuner 2015).

Abbreviations: ADI‐R, autism diagnostic interview‐revised; ADOS, autism diagnostic observation schedule; BOSCC, brief observation of social communication change; RRB, restricted and repetitive behaviors; SRS, social responsiveness scale (Bölte et al. 2008).

### 1.2 Procedure

**FIGURE 1:**
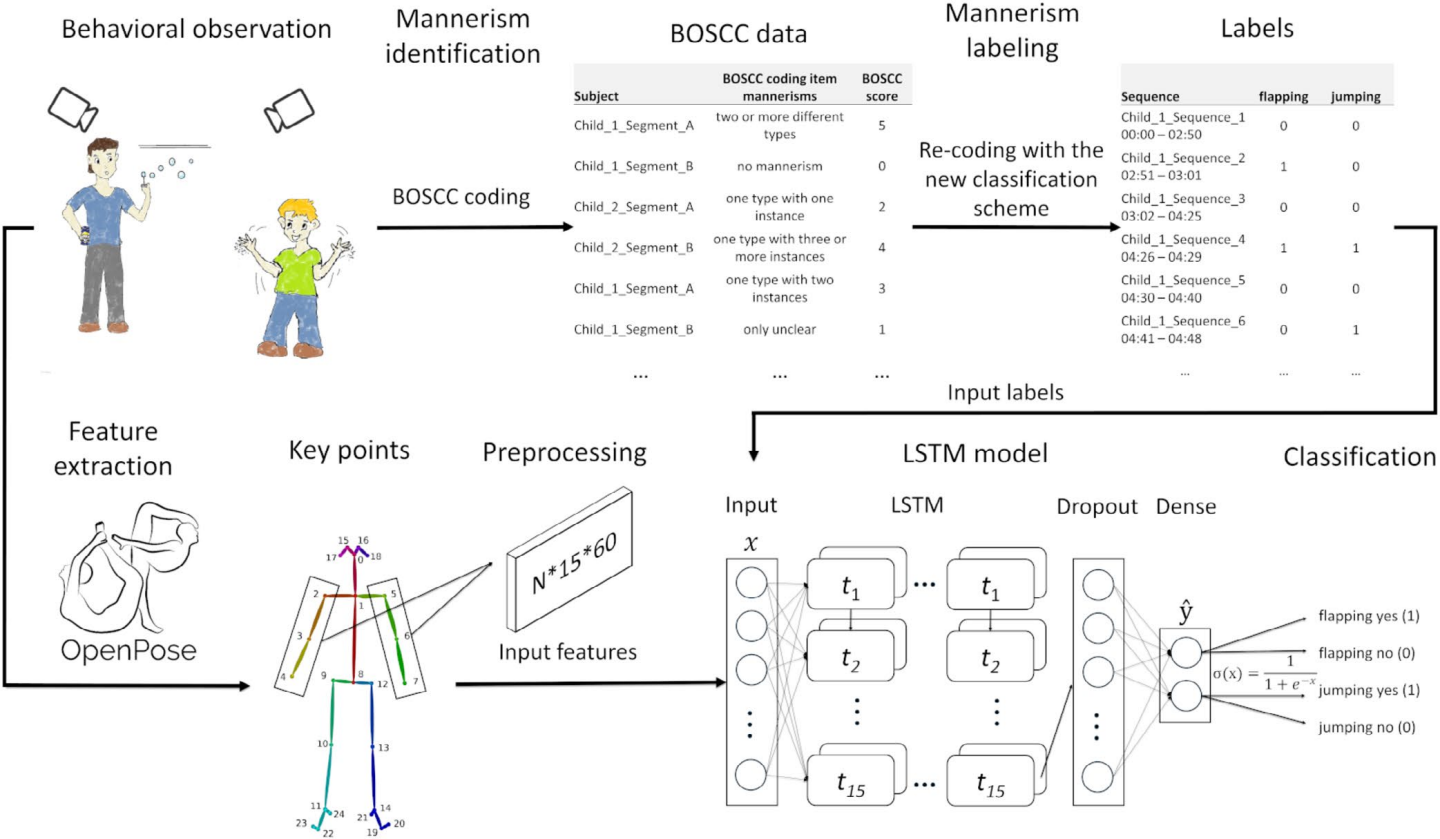
Study workflow: The combination of OpenPose feature extraction and LSTM model for multi‐label classification of mannerisms: BOSCC = Brief Observation of Social Communication Change. OpenPose = Algorithm used for feature extraction of body key points. *t*_1_−*t*_15_ = 15 frames (timesteps). Input, LSTM, dropout, and dense = layers in the network. *σ* = Sigmoid activation in the dense layer.

We analyzed a 12‐min video sequence of a free‐play situation with an experimenter for all participants, conducted as part of the clinical trial (see Figure 1 for a study workflow). Each video sequence was divided into two 6‐min segments. In each segment, the tester encouraged a child to play with a different set of toys for 4 min followed by 2 min of bubble play according to the brief observation of sSocial communication change (BOSCC), research version of December 11, 2017 (Grzadzinski et al. 2016). A caregiver was present in the room and encouraged to remain passive. We recorded all videos using two Full HD (1920 × 1080) cameras positioned at opposite viewpoints to capture the entire room (see Figure S1 in Supporting Information for the testing room setup). The videos were pre‐coded using the BOSCC coding procedure, which identified autistic mannerisms on a per‐segment level. Next, we developed a classification scheme to assign mannerism labels to each occurrence of mannerism within a segment (see “re‐coding” below). The classification scheme defined the start and end of each occurrence. These single occurrences of mannerisms were labeled as a ground truth by two raters using Interact (Mangold 2020). The labeled sequences were subsequently used in supervised learning of the ML algorithm. We applied the OpenPose algorithm for feature extraction of human skeleton key points from the video sequences (see “feature extraction” below). These features were then reshaped into 15‐frame sequences (see “preprocessing”) and utilized as input for an LSTM network for multi‐label classification of autistic mannerisms.

### 1.3 Coding

#### 1.3.1 Pre‐Coding (BOSCC)

The BOSCC (Grzadzinski et al. 2016) is a coding scheme that had been developed to assess changes in ASD core symptoms over time based on a video‐recorded behavioral observation. The BOSCC total score includes the 8‐item social communication (SC) subscale and the 4‐item Repetitive Behaviors (RRB) subscale. All videos were pre‐coded using the BOSCC coding scheme. We focused on the RRB‐item “Hand and Finger or Complex Body Mannerism” to identify the occurrence of mannerisms. The pre‐coding served as a starting point for labeling sequences based on clinical expertise but was not suitable as labels for an ML algorithm due to several reasons: (1) a lack of standardization in behavior labeling, (2) BOSCC ratings are aggreged per video segment and not based on single occurrences of mannerisms, and (3) the absence of defined sequences start and stop points. This was addressed through re‐coding, as described in the next section.

#### 1.3.2 Re‐Coding Classification Scheme for Mannerisms

We developed a classification scheme based on the BOSCC coding to label different types of mannerisms (Table 3). This provided the ground truth for supervised learning. The new classification scheme allows for the assignment of multiple labels (e.g., “flapping” and “jumping”) per sequence, which are utilized in a multi‐label classification algorithm (see “multi‐label classification” below). A mannerism was labeled as unclear when its presence was not definitive according to BOSCC coding. For instance, a child could show flapping with one arm atypically or react to a fly on the hand. We also coded the start and end of each sequence with timing labeled per second. Sequence lengths during re‐coding ranged from 1 s (e.g., brief flapping) to 6 min (entire segments without mannerisms).

**TABLE 3:** Mannerism classification scheme.

Mannerism	Description	Unclear	Specifier
Flapping	Flapping with arms	Yes/no	Right/left
Clapping	Clapping hands together (or on the body)	Yes/no	Right/left
Jumping		Yes/no	
Hand/finger	Fast hand/finger movements or odd hand/finger posture	Yes/no	Right/left, fast/still
Legs	Movements with legs	Yes/no	Right/left
Tiptoeing		Yes/no	Heel gait
Body	Movements/postures without jumping or flapping (incl. body rocking)	Yes/no	

#### 1.3.3 Inter‐Rater Reliability (IRR) of Re‐Coded Mannerisms

We developed an individual consensus training for re‐coding using the new classification scheme, based on the recommended BOSCC training. A learning rater and an experienced rater had to meet consensus criteria. Consensus was reached when they differed by zero points for 80% of single mannerism ratings across three consecutive videos (or six segments) and by no more than three points on the total score or unclear items. Two raters performed coding in the present study. Rater 1 (the first author) was an experienced BOSCC coder, trained by co‐author J.K.C., who received training from the BOSCC development team. Rater 2 had no prior experience with the BOSCC or autistic children. Consensus between Rater 1 and Rater 2 was reached after six videos (12 segments). IRR was calculated from 8 segments (4 randomly selected videos) using the intraclass correlation coefficient (ICC; Two‐way, mixed effects). Consensus ratings covered a total of 10 videos (19%) based on previous BOSCC studies (Frost et al. 2019; Grzadzinski et al. 2016; Kim et al. 2019; Kitzerow et al. 2016; Pijl et al. 2018). ICCs ranged from excellent (Koo and Li 2016) for items clapping (1.0) and jumping (1.0), good for flapping (0.89 [0.53, 0.98]), to moderate for the total score (0.64 [−0.05, 0.92]). Hand/finger mannerisms had poor reliability (0.16 [−0.55, 0.75]) and were excluded from the analysis. Other mannerisms did not appear in the IRR videos and were excluded from the analysis.

### 1.4 Machine Learning

#### 1.4.1 Feature Extraction

The OpenPose algorithm (Cao et al. 2018) was used to extract features on a Windows PC with a low‐end graphics card (NVIDIA GeForce GTX 1650; GPU). OpenPose is an open‐source, real‐time, multi‐person computer vision framework. It uses a multi‐stage CNN architecture to extract human skeleton key points. OpenPose can process single images, video files, or real‐time video feeds and is widely used for pose recognition (Fourie and van der Haar 2020; Yadav et al. 2019). We custom‐built OpenPose using CMake‐GUI. The GPU was used to speed up the process in “GPU_MODE” with Cuda Version 11.1. Custom parameters (flags) included setting “—net_resolution” to “1×288” Output was saved as JSON files, generating one file for each video frame. We applied the predefined Body25 model, which extracts 25 body key points with *x* and *y* coordinates along with accuracy values. The OpenPose Hand model was not applied due to hardware limitations. A cluster or online GPU was not used because of data privacy concerns with the video files.

#### 1.4.2 Preprocessing

All preprocessing and analysis scripts are available on GitHub: https://github.com/chrstnlmlr/ml_asd. We used minimal preprocessing to highlight the objective and semi‐automated classification. Python version 3.9 was applied for all preprocessing tasks. Since OpenPose lacks a built‐in person‐tracking function, we used a Python script to track individuals across JSON files (Barami 2020). The script calculates the center of mass for each person in each frame. It then assigns the same person ID to the individual with the nearest center of mass compared to the previous frame. The script usually detects three people: the child, the tester, and the caregiver. Occasionally, a person walking in and out of the camera's view is marked as a “new person.”

The modified JSON files were then loaded into a data frame. We extracted 6 key points (shoulder, elbow, and wrist for both arms). Key points with accuracy below 0.6 were discarded to minimize misidentifications (Cook et al. 2019). Coordinates were normalized from Full HDTV resolution (1920 × 1080) to values between 0 and 1, enabling compatibility with other video formats. Sequences between 1 s (25 frames) and 6 min were divided into 15‐frame sequences for training and testing. We kept sequences of at least 15 frames to preserve the time‐series structure. The coordinates for the 6 key points were aligned side by side for all individuals, ensuring detection of mannerisms in a multi‐person environment with up to 5 persons. We assumed that testers and caregivers did not exhibit any mannerisms. The key points from both cameras were used as input in the sense of data augmentation. This preprocessing resulted in input for the LSTM model with the following structure: number of sequences (*N*) * number of frames per sequence (15) * number of features (5 persons × 2 coordinates × 6 key points = 60).

#### 1.4.3 Sequence Selection for Training and Testing

Mannerisms occurred in 2% of the preprocessed dataset. For training and testing, we focused only on scenes with flapping and jumping (Table 4). Clapping was excluded as it mostly occurred alongside other mannerisms. Other mannerisms were excluded as described earlier. The final dataset included 930 sequences with flapping and jumping. To balance the dataset, we randomly selected an equal number of sequences with no mannerisms. The sequences without mannerisms were drawn from all 52 children. This resulted in a total of 1890 sequences with a sequence length of 15 frames for training and testing the multi‐label classification algorithm.

**TABLE 4:** Mannerisms in the dataset of *n* = 52 autistic preschoolers.

Mannerism	After preprocessing and sequence selection (15 frames)		Before preprocessing and sequence selection (min 15 frames)	
	Sequences	Children	Sequences	Duration^a^
	*n* (%)	*n* (%)	*n* (%)	s (*M*, SD)
Flapping (*M*)	819 (88.06)	22 (100.00)	188 (86.24)	0.6–17.4 (4.04, 3.27)
Clapping (*M*)	124 (13.33)	2 (9.09)	19 (8.72)	2.4–11.4 (5.62, 0.48)
Jumping (*M*)	390 (41.94)	10 (45.45)	95 (43.58)	0.6–12.0 (3.85, 2.76)
Hand (*M*)	205 (22.04)	5 (22.73)	34 (15.60)	2.4–17.4 (6.23, 3.82)
Leg (*M*)	12 (1.29)	3 (13.64)	5 (2.29)	1.2–3.0 (2.28, 0.99)
Tiptoeing (*M*)	134 (14.41)	4 (18.18)	15 (6.88)	1.2–17.4 (9.24, 4.56)
Body (*M*)	9 (0.97)	1 (4.55)	2 (0.92)	3.0–3.0 (3.00, 0.00)
Flapping + jumping	195 (20.97)	1 (4.55)	50 (22.94)	1.2–9.6 (3.55, 2.35)
Flapping	303 (32.58)	2 (9.09)	84 (38.53)	0.6–13.8 (2.95, 2.54)
Jumping	36 (3.87)	1 (4.55)	17 (7.80)	0.6–6.0 (2.22, 1.54)
Mannerism	930 (100.00)	22 (100.00)	218 (100.00)	0.6–17.4 (3.93, 3.16)

*Note*: Sequences denoted with an (*M*) may contain multiple mannerisms in parallel. All others count only the named mannerisms or combinations, while no other mannerism is present.

^a^ Duration was calculated with 25 frames per second. Sequences shorter than 15 frames were discarded at this stage.

#### 1.4.4 Multi‐Label Classification

In binary classification, sequences are assigned one label from two classes: “mannerism” or “no mannerism.” In multi‐class classification, sequences receive one label from more than two classes: “no mannerism,” “flapping,” or “jumping.” In this study, we used multi‐label classification. This approach assigns zero or more labels to each sequence: “no mannerism,” “flapping,” “jumping,” or “flapping and jumping.” This method better reflects real‐world behavioral observations.

#### 1.4.5 Model

**FIGURE 2:**
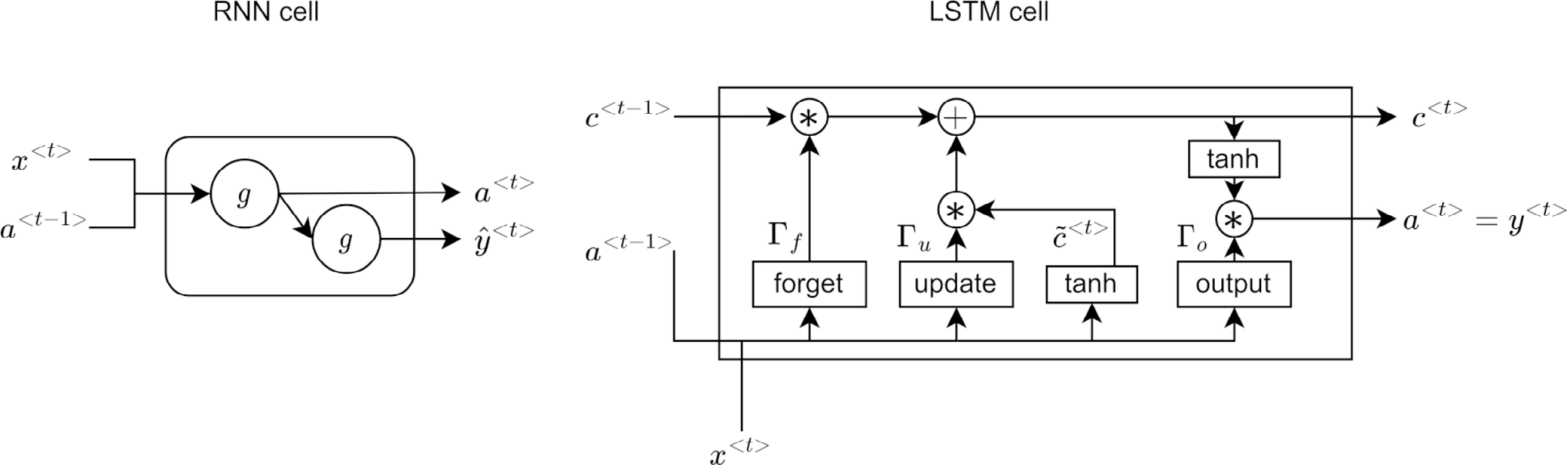
Comparing the cells of RNN and LSTM: RNN cell = left side shows a cell in a recurrent neural network. LSTM cell = right side shows a long short‐term memory network cell. *Element‐wise product. tanh, hyperbolic tangent.

We used a neural network structure with LSTM, dropout and dense layers (Figure 1). LSTMs are a type of Recurrent Neural Network (RNN), which apply loops and hidden states (Figure 2, left), allowing them to process time‐series data like video sequences. In an RNN, $x^{<t>}$ represents the input, $a^{<t>}$ (Equation 1) is the hidden state, and ${ŷ}^{<t>}$ (Equation 2) is the output at timestep *t*. The activation function is denoted by *g*, with weights and biases represented as *W* and *b*. The updates are computed sequentially, where $a^{0}$ is a zero vector:

(1) $$a^{<t>}=g\left(W_{a}\left[\;a^{<t-1>},x^{<t>}\right]+b_{a}\right)$$

(2) $${ŷ}^{<t>}=g\left(W_{y}\;a^{<t>}+b_{y}\right)$$

However, RNNs tend to forget earlier inputs in longer sequences due to vanishing gradients, which hinders training of long‐term dynamics (Hochreiter and Schmidhuber 1997). LSTMs address this with additional gates: the update gate ($\Gamma_{u}$), the forget gate ($\Gamma_{f}$), the output gate ($\Gamma_{o}$), and the cell state ($c^{<t>}$) (Figure 2, right). LSTM updates for timestep t are calculated as follows (* represents an element‐wise product):

(3) $$\Gamma_{u}=\sigma \left(W_{u}\left[\;a^{<t-1>},x^{<t>}\right]+b_{u}\right)$$

(4) $$\Gamma_{f}=\sigma \left(W_{f}\left[\;a^{<t-1>},x^{<t>}\right]+b_{f}\right)$$

(5) $$\Gamma_{o}=\sigma \left(W_{o}\left[\;a^{<t-1>},x^{<t>}\right]+b_{o}\right)$$

(6) $${\tilde{c}}^{<t>}=\tanh\left(W_{c}\left[a^{<t-1>},x^{<t>}\right]+b_{c}\right)$$

(7) $$c^{<t>}=\Gamma_{u}*{\tilde{c}}^{<t>}+\Gamma_{f}*c^{<t-1>}$$

(8) $$a^{<t>}=\Gamma_{o}*c^{<t>}$$

Equation (3) determines the new input (update $\Gamma_{u}$) to store in the cell state. Equation (4) computes the information to forget (forget gate $\Gamma_{f}$) from the cell state. Equation (5) calculates the output (output gate $\Gamma_{o}$), which transfers information from the cell state to the hidden state $a^{<t>}$. The memory cell $c^{<t>}$ (Equation 7) is the sum of the previous memory $c^{<t-1>}$, modulated by the forget gate, and the current input with previous hidden state ${\tilde{c}}^{<t>}$ (Equation 6), modulated by the input gate. These additional components enable LSTMs to learn complex, long‐term temporal relationships (Donahue et al. 2015). LSTMs use two activation functions: the sigmoid function ($\sigma \left(x\right)=\frac{1}{1+e^{-x}}$), which scales inputs between 0 and 1, and the hyperbolic tangent ($\tanh\left(x\right)=\frac{e^{x}-e^{-x}}{e^{x}+e^{-x}}$), which scales inputs between −1 and 1.

#### 1.4.6 Nested Cross‐Validation

**FIGURE 3:**
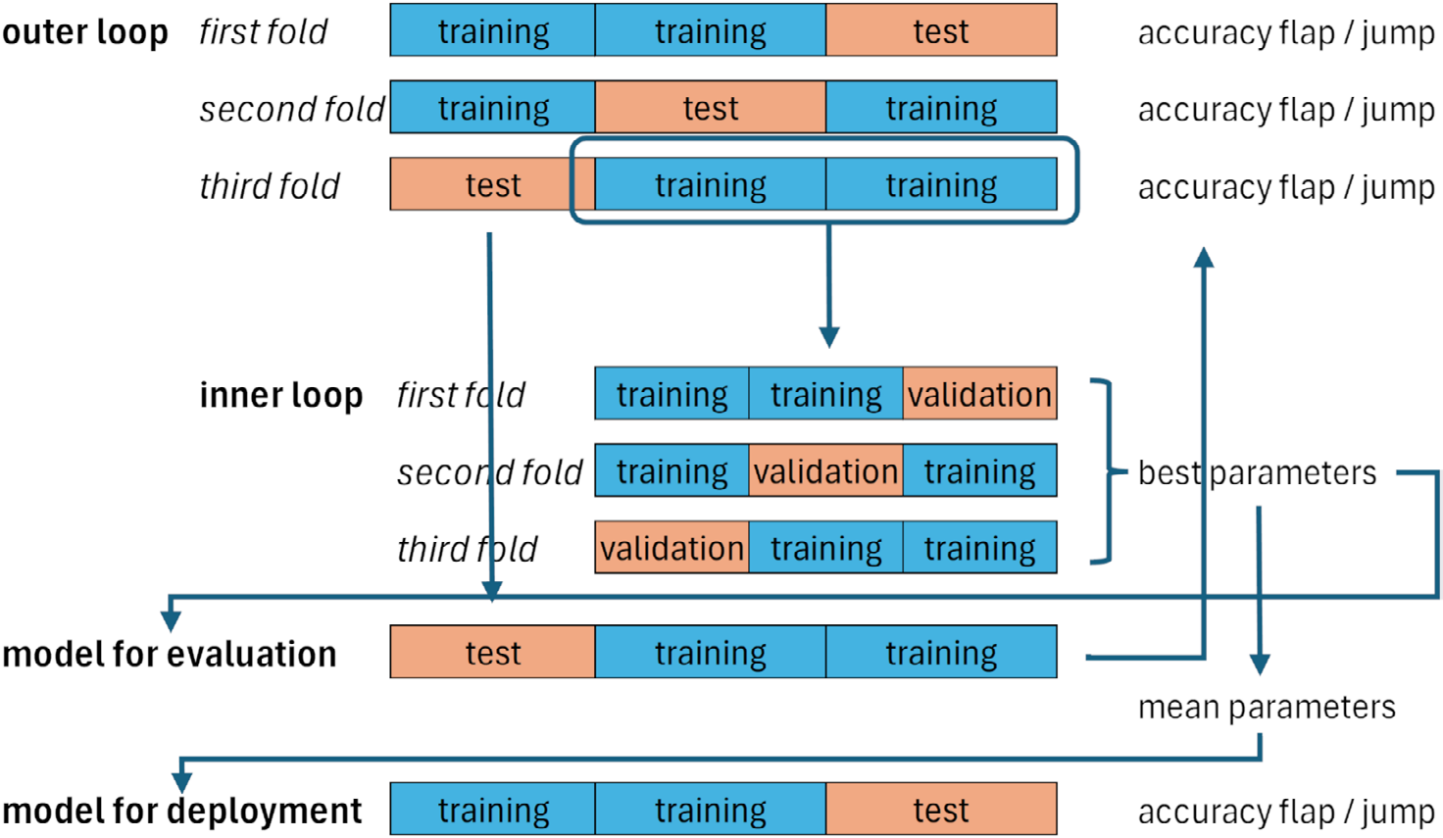
Nested 3‐fold cross‐validation process. Outer loop for model evaluation. Inner loop for hyperparameter tuning.

We implemented a nested 3‐fold cross‐validation to evaluate the model on unseen data, providing a reliable estimate of its generalization performance. The outer loop was used for model evaluation, and the inner loop was used for hyperparameter tuning (Figure 3).

**FIGURE 4:**
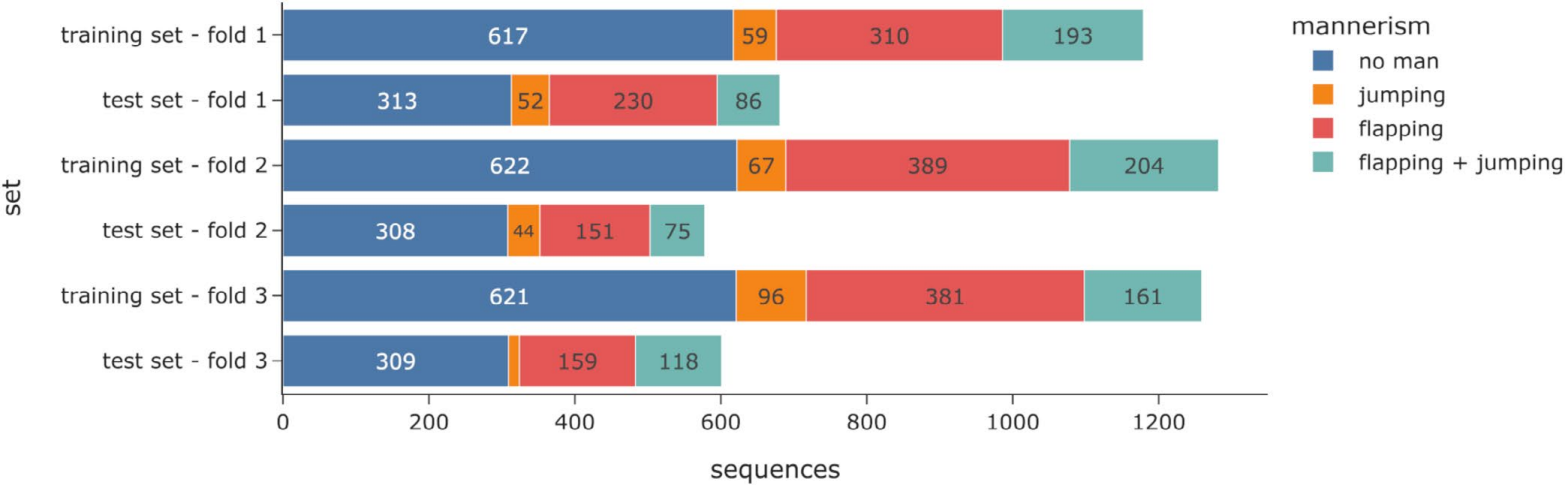
Distribution of mannerisms in outer loop of nested 3‐fold cross‐validation.

In the outer loop, we used Scikit‐Learn's StratifiedGroupKFold (Pedregosa et al. 2011) to create multiple training and test splits (Figure 4). This preserved the distribution of multi‐labels while ensuring no subject was split between folds (subject‐wise split). By separating children between the training and test sets, we emphasized the clinical applicability of our approach.

In the inner loop, hyperparameter tuning was conducted using only the training data from each outer fold. This ensured that the test set remained untouched during the tuning process. After tuning, the model was retrained on the entire training set using the best hyperparameters, and performance was evaluated on the outer test set (Figure 3). This process was repeated for each fold, providing an unbiased estimate of the model's performance. For deployment, we trained a model using the means of the best hyperparameters across all folds on the full dataset.

To compare approaches, we reran the nested cross‐validation using a random split instead of a subject‐wise split. In the random split, the same child could appear in both the training and test sets, which has been applied in most previous studies but contradicts diagnostic applicability.

#### 1.4.7 Hyperparameter Tuning

Hyperparameter tuning is commonly applied with cross‐validation to prevent overfitting and improve model performance (Feurer and Hutter 2019). We used the Keras Hyperband tuner for tuning (Li et al. 2017), as there are no established LSTM hyperparameters for mannerisms classification. The search space for LSTM units was defined for three layers with values ranging from 128 to 512 (step size 128). The input LSTM layer was fixed with an input shape of (15, 60), where 15 frames represent the timesteps and 60 is the number of features.

After the last layer, a dropout layer was applied with a dropout rate between 0.0 and 0.15 (step 0.05), followed by a dense layer with two neurons for multi‐label classification. The Adam optimizer was chosen with a learning rate of 0.0003. The batch size was tuned to 16 or 32, and the loss function was “binary_crossentropy” for multi‐label classification. All searches were run for 1000 epochs on a High‐Performance Computing cluster since the preprocessed input data (i.e., skeletal tracking) contained no personally identifiable information.

Hyperparameters were tuned during nested cross‐validation. Table 5 shows the best hyperparameters found using the Keras Hyperband tuner and the mean of the best hyperparameters, which we used to train a final model for deployment.

**TABLE 5:** Hyperparameter from Keras Hyperband tuner for subject‐wise split.

Hyperparameter	Fold 1	Fold 2	Fold 3	Mean
units_layer_1	256	256	128	213
units_layer_2	512	384	384	427
units_layer_3	384	512	512	469
dropout_rate	0.1	0.1	0.05	0.083
batch_size	32	16	32	27

*Note*: mean = mean of folds 1 to 3 used for deployment model.

#### 1.4.8 Evaluation Metrics of Model Performance

Table 6 presents a confusion matrix for a binary classification. The most common evaluation metric is accuracy (9), representing the proportion of correctly classified cases out of the total (Thabtah 2019). Recall (10) or sensitivity, measures the correctly classified mannerisms (true positive rate), while specificity (11) indicates the true negative rate. Precision (12) determines how many of the predicted positive examples are positive, and the *F*1 score (13) is the harmonic mean of precision and recall. Evaluation metrics (9), (10), (11), (12), (13) were used to evaluate the performance of the LSTM model for multi‐label classification of mannerisms in videos.

(9) $$\text{accuracy}=\frac{\mathrm{TP}+\mathrm{TN}}{P+N}$$

(10) $$\text{recall or sensitivity}=\frac{\mathrm{TP}}{\mathrm{TP}+\mathrm{FN}}$$

(11) $$\text{specificity}=\frac{\mathrm{TN}}{\mathrm{TN}+\mathrm{FP}}$$

(12) $$\text{precision}=\frac{\mathrm{TP}}{\mathrm{TP}+\mathrm{FP}}$$

(13) $$F1\;\text{score}=\frac{2\mathrm{TP}}{2\mathrm{TP}+\mathrm{FP}+\mathrm{FN}}$$

**TABLE 6:** Confusion matrix.

	Predicted negative (PN)	Predicted positive (PP)
Actual negative (N)	True negative (TN)	False positive (FP)
Actual positive (P)	False negative (FN)	True positive (TP)

## 2 Results

The subject‐wise split models learned to classify different types of mannerisms, a combination of mannerisms, or the absence of mannerisms in the video sequences. We calculated results separately for each label: flapping and jumping. Table 7 presents the evaluation metrics from cross‐validation for the subject‐wise split models.

**TABLE 7:** Evaluation metrics for multi‐label classification with a subject‐wise split dataset.

Metrics	Flapping	Jumping	Macro average	Macro average (deployment)
Accuracy	0.647 (0.607–0.669)	0.758 (0.717–0.809)	0.702	0.705
Recall	0.390 (0.261–0.484)	0.101 (0.034–0.226)	0.245	0.305
Specificity	0.843 (0.827–0.871)	0.932 (0.888–0.974)	0.887	0.852
Precision	0.647 (0.496–0.740)	0.307 (0.090–0.714)	0.477	0.470
*F*1 score	0.485 (0.342–0.574)	0.151 (0.052–0.343)	0.318	0.362

*Note*: Each cross‐validation set contains 1860 sequences (Figure 4) with a sequence length of 15 frames. Minimum and maximum values from cross‐validation are shown in brackets. The macro average represents the model's overall performance across both classes. “Deployment” refers to the results of the final model trained with the mean of the best hyperparameters.

These results demonstrate that the subject‐wise split model could detect mannerisms in videos from unknown individuals. Accuracy was higher for detecting jumping than flapping, suggesting more correct predictions (true positives and true negatives) for jumping. The higher specificity for jumping also indicates more correct negative predictions. However, precision, recall, and *F*1 scores were higher for flapping, suggesting the model was better at identifying true positives (recall) and had fewer false positives (precision) for flapping. In one cross‐validation fold, recall, precision, and *F*1 score for jumping were nearly zero, indicating the model had difficulties detecting jumping in that fold. The models successfully differentiated between frequent mannerisms in videotaped behavioral observations.

## 3 Discussion

We developed a ML pipeline using the CNN‐based OpenPose algorithm for feature extraction and an LSTM network for multi‐label classification of mannerisms from pre‐recorded video data. The algorithm successfully detected clinically relevant mannerisms in autistic children within a multi‐person environment of naturalistic behavioral observations, distinguishing these from non‐RRB behaviors. Our results with subject‐wise split models showed that mannerisms could be identified in new, unknown children, highlighting the diagnostic potential of this approach. All models were able to simultaneously label different mannerisms in video sequences, achieving high accuracy (0.702). We also retrained a deployment model, using the means of the best hyperparameters from cross‐validation, which achieved almost the same accuracy (0.705). Previous studies relied on random split evaluation, leading to overfitting to the subjects and limiting comparability to subject‐wise evaluation (Cook et al. 2019; Vyas et al. 2019; Zhang, Tian, et al. 2021). Therefore, the present study establishes a baseline under a more robust evaluation strategy.

We advanced the previous LSTM approach by (1) applying multi‐label classification to identify different mannerisms within a single model. Our results showed that detecting multiple mannerisms simultaneously is possible, unlike earlier studies that labeled each sequence with only one category (multi‐class labeling). Additionally, we (2) introduced a clinically driven classification scheme for mannerisms. Previous deep learning approaches used labels with binary arguments: “typical” versus “atypical” (Cook et al. 2019; Vyas et al. 2019) or labeled with “sit,” “stand,” “shake hands,” “shake the body,” and “squat” (Zhang, Tian, et al. 2021). In contrast, we developed a clinically driven labeling based on the BOSCC coding. All children were diagnosed with ASD using gold standard procedures from a multicenter study. We achieved reliable inter‐rater agreement for the two most frequent mannerisms, “flapping” and “jumping” (Table 4). In addition, a key feature of our analysis was (3) splitting the data by subjects. This allowed us to detect mannerisms in unknown children, enhancing the clinical relevance of the algorithm.

We conclude that machine learning‐based classification of mannerisms holds promise for enhancing objective diagnostic procedures in future clinical practice. Our automated algorithm classified mannerisms in autistic children with high accuracy, using naturalistic, videotaped behavioral observations. Applying this algorithm could improve the current manual coding of mannerisms, which suffers from low reliability (Carruthers et al. 2021). We present an easy‐to‐implement, open‐source solution that could be scaled to future studies with low coding effort. The algorithm could also be applied to existing video recordings in many clinical institutions. We envision a productive system, where our model supports manual coding by automatically annotating videos (e.g., “likely flapping” and “likely jumping”). This could also enhance agreement between coders, particularly in intervention studies (see Videos S1 and S2 in Supporting Information for a demonstration of the classification output).

For further research, we recommend including more key points in the training process, as classifying jumping might improve by incorporating leg movements. Similar movements should also be considered during training, as our model was handicapped when jumping sequences included other movements like clapping that more closely resemble flapping. Additionally, the limited number of jumping‐only sequences (36 in total, Table 4) hindered the classification of jumping. Furthermore, only 1 child out of 52 exhibited solely jumping, which affected model performance in the subject‐wise split. Future ML studies—especially those without BOSCC pre‐coding—could aim for a higher double‐coding rate (compared to the 19% in our study) to further enhance reliability. Finally, expanding the dataset with more labeled training data could also improve performance. A larger sample size would provide more data for subject‐wise splits, increasing clinical applicability and enabling multi‐label training with additional labels like “clapping” or “legs” Improved performance might also be achieved using a high‐end graphics card, allowing processing at a higher accuracy setting in feature extraction and possibly enabling hand recognition.

In conclusion, our open‐source models (https://github.com/chrstnlmlr/ml_asd) could serve as a foundation for automated coding of behavioral observations. We propose that low inter‐rater reliabilities in manual coding could be addressed through ML‐based video annotations. As a next step toward fully automated coding could be an evaluation at the level of entire sequences, segments, or even full videos could be conducted. However, the low prevalence of observed mannerisms (only 2% of sequences in our data) remains a challenge for future studies. The holistic approach to identifying autistic behavior in comprehensive ADOS videos, as demonstrated by Kojovic et al. (2021), also yielded lower accuracy in detecting mannerisms. Combining their holistic method with our mannerism‐specific algorithm might address specific gaps in autism diagnostics, which still rely on manual coding. Finally, future work could explore 3D‐CNN ResNets, which have shown promising results in ASD detection and action recognition tasks, as a potential alternative to LSTMs for capturing both spatial and temporal aspects of manneristic behavior (Zhang, Wang, et al. 2021).

## Author Contributions

**Christian Lemler:** conceptualization, software, data curation, formal analysis, data collection, data preprocessing, data analysis, writing – original draft, visualization. **Nico Bast:** conceptualization, supervision, writing – review and editing. **Solvejg K. Kleber**, **Leonie Polzer**, and **Naisan Raji:** data collection. **Simeon Platte:** data analysis. **Janina Kitzerow‐Cleven** and **Ziyon Kim:** project management for the associated clinical trial. **Christine M. Freitag:** project administration, funding acquisition, conceptualization. All authors read and approved the final manuscript.

## Consent

The caretakers of all participants have signed a declaration of consent to participate in the study.

## Conflicts of Interest

C.M.F. and J.K.‐C. receive royalties for books on ASD and ADHD. N.B. receives royalties for lecturing at institutes for training in psychotherapy. C.M.F. and N.B. receive research funding from the German Research Foundation (DFG). C.M.F. further receives funding from the German Ministry of Science and Education and the European Commission.

## Supporting information


**Figure S1.** Testing Room with approximate measures. Measures could vary slightly through the four different study sites: Frankfurt (n=37), Augsburg (n=12), Würzburg (n=2), Dresden (n=1). The height of the cameras is approximately 1.70m.

**Video S1.** This video demonstrates a sequence with mannerism from the BOSCC (Brief Observation of Social Communication Change). Body key points were extracted using the OpenPose algorithm. The overlay “likely flapping” represents true positive predictions from the final model developed in this study. Since no independent test dataset was available, the demonstration uses data from the cross‐validation process. The prediction code is available on GitHub: https://github.com/chrstnlmlr/ml_asd.

**Video S2.** This video demonstrates a sequence without mannerisms from the BOSCC (Brief Observation of Social Communication Change). Body key points were extracted using the OpenPose algorithm. The absence of overlays (e.g. “likely flapping”) represents true negative predictions from the final model developed in this study. Since no independent test dataset was available, the demonstration uses data from the cross‐validation process. The prediction code is available on GitHub: https://github.com/chrstnlmlr/ml_asd.

## Data Availability

The code and the ML models generated during the current study are available in the GitHub repository, https://github.com/chrstnlmlr/ml_asd. The original video files are not publicly available due to data privacy agreements. The datasets used for training and testing the models are available from the first author upon reasonable request.
