# Supplementary figures and images for: Semi‐Automated Multi‐Label Classification of Autistic Mannerisms by Machine Learning on Post Hoc Skeletal Tracking
Source: Autism Res. 2025 Mar 14;18(4):833–44. doi: 10.1002/aur.70020 (PMC12015794; doi:10.1002/aur.70020)

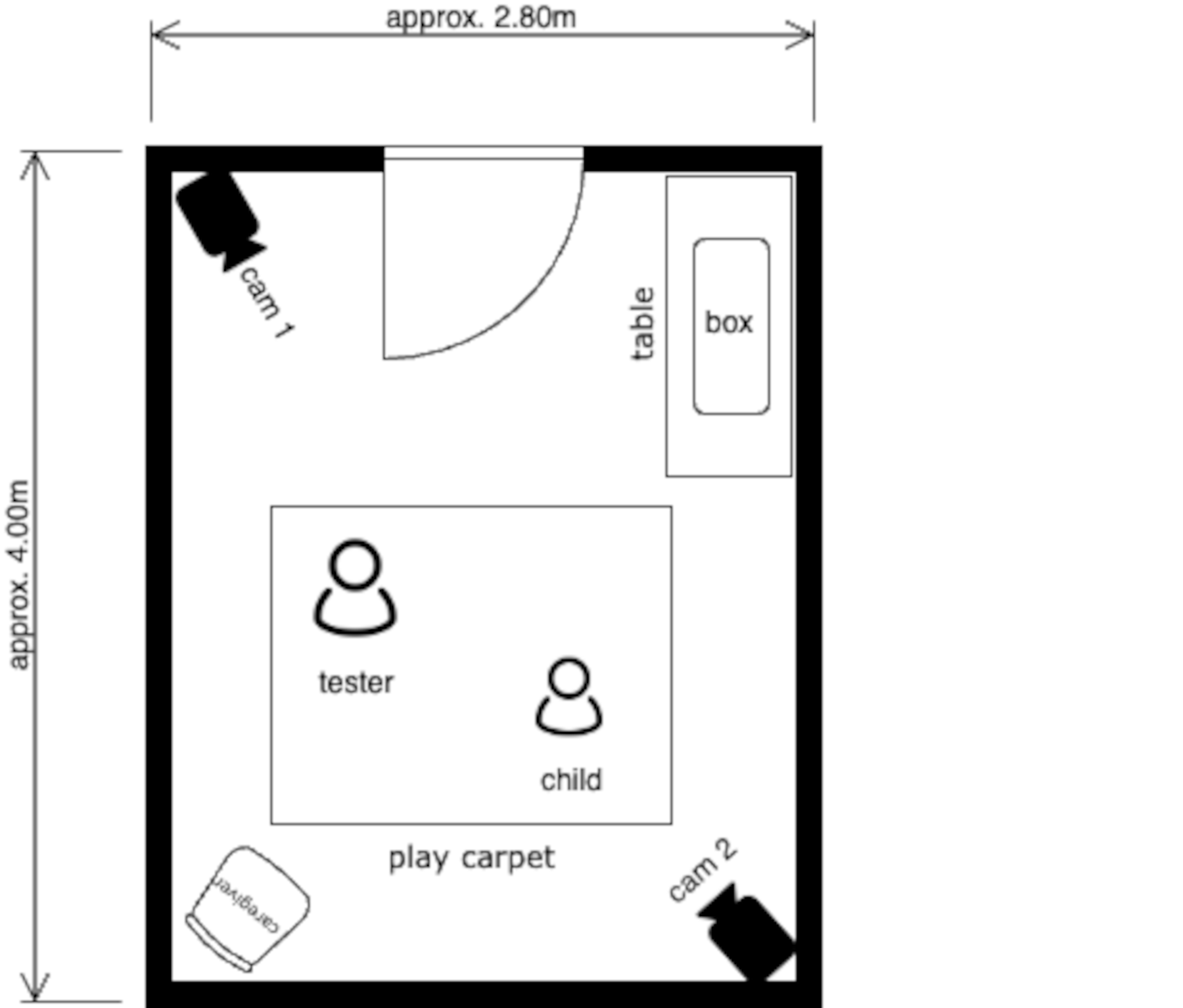

Supplement: Supplementary file 1 — Figure S1. Testing Room with approximate measures. Measures could vary slightly through the four different study sites: Frankfurt (n=37), Augsburg (n=12), Würzburg (n=2), Dresden (n=1). The height of the cameras is approximately 1.70m. [file AUR-18-833-s001.png]
